# Differences in Risk Factors for Rotator Cuff Tears in Young Versus Old Individuals

**DOI:** 10.7759/cureus.64259

**Published:** 2024-07-10

**Authors:** MaKenzie Chambers, Puvin Dhurairaj, Aditya Joshi, Manisha Koneru, Pietro M Gentile, Catherine Fedorka

**Affiliations:** 1 Orthopedics, Cooper Medical School of Rowan University, Camden, USA; 2 Orthopedics, Cooper University Hospital, Camden, USA; 3 Neurointerventional Surgery, Cooper Medical School of Rowan University, Camden, USA; 4 Rheumatology, Cooper University Health Care, Camden, USA

**Keywords:** full thickness, bmi, age, risk factor, rotator cuff tear

## Abstract

Background

The etiology of rotator cuff tears is thought to be multifactorial with current literature that varies with regard to identifiable risk factors. The purpose of this retrospective review was to identify risk factors for full-thickness rotator cuff tears and determine whether they differ in young versus older individuals.

Methods

To determine the presence or absence of a rotator cuff tear, 1,561 patients with a shoulder MRI were reviewed. If a tear was present, it was further classified into a partial or full-thickness tear. Demographic variables and clinical data were collected and analyzed with a two-sided Student’s t-test or Wilcoxon rank sum test for continuous variables and a Chi-square test or Fisher’s exact test for categorical variables. Age and BMI were dichotomized using receiver operator curves.

Results

Charlson Comorbidity Index, age, BMI, sex, race, and work status were all factors that variably affected a patient's risk of experiencing a rotator cuff tear, with different factors carrying more influence on outcomes within those who are older versus those who are younger. Gender and race were found to differ as risk factors between young and older individuals.

Conclusion

We were able to identify risk factors overall associated with increased odds of sustaining a full-thickness rotator cuff tear. Our analyses also showed differences in the effect of gender and race as risk factors between young and older patients with rotator cuff tears. This finding may aid clinicians in counseling patients on more specific risks for their given age.

## Introduction

Rotator cuff tears (RCTs) are a potentially debilitating condition that can significantly impact a person's quality of life. The etiology of RCTs arises from a complex interplay of factors, with multiple variables theorized to contribute to an individual's risk of developing a tear [1-3]. Current literature aims to identify possible risk factors associated with an increased probability of developing an RCT, poor patient outcomes after repair, and postoperative complications [1,2,4,5]. Age is consistently reported as a risk factor for RCTs, with a greater prevalence of tears observed within older patients [2]. Lifestyle factors, such as smoking, have also been linked to an increased risk of tear as the chemicals in cigarettes have been noted to accelerate degeneration and weaken the rotator cuff [6]. Additionally, BMI, occupation, activity level, upper extremity muscle imbalance, vaccine injection, and underlying medical conditions such as diabetes are other factors that have been identified as potential risk factors for the development of an RCT [1-3,5,7-9]. Though recent studies have reported data that support an increased predisposition for RCT in patients with one of these factors, there is variation as other studies have demonstrated opposite effects [1,5].

In addition to the variation among identifiable risk factors for RCT, there is also a question as to whether these risk factors carry the same impact at all ages or differ between younger and older populations. One study by Watanabe et al. reported that trauma was a distinguishing factor between patients younger than 64 and those older than 64 in predicting RCTs [10]. In contrast, other factors examined, such as critical shoulder angle, did not differ. While this study revealed a difference in risk factors for RCTs among younger and older patients, additional literature examining these relationships between age groups is scarce. Because of this, the primary aim of this study is to investigate the potential risk factors associated with RCTs and determine whether differences exist between patients in a younger age group and those in an older age group. We hypothesize that our two cohorts will have significant differences in identifiable risk factors when stratified by age. Due to the complex interplay of factors that contribute to increasing the risk of RCTs, the findings of this study will be clinically significant for orthopedic surgeons as they counsel and educate patients presenting with musculoskeletal complaints. By identifying the modifiable risk factors relevant to various age groups, physicians can draw attention to the value of preventive and protective measures to potentially mitigate the risk of a tear.

## Materials and methods

This IRB-approved study queried our institution’s electronic medical record system utilizing the CPT codes 73221, 73222, and 73223 for upper extremity joint MRI to identify all shoulder MRIs completed at level 1 trauma center/tertiary referral center from January 1, 2019 to December 31, 2021. A total of 1,561 patients, 18 years or older, who had an MRI of the shoulder met the inclusion criteria. Level of activity and/or participation in athletics was not considered in our inclusion/exclusion criteria as all patients with a shoulder MRI were included. Any patients missing demographic and comorbidity data were excluded.

A retrospective review of these patient charts was performed to collect patient demographic data (gender, race, BMI, age, sex, Charlson Comorbidity Index (CCI)) and clinical characteristics (size of RCT, laterality, surgical intervention). Additional data on social factors were also collected (substance use and occupational status). Statistical analysis was performed using a two-sided Student’s t-test or Wilcoxon rank sum test for continuous variables and a Chi-square test or Fisher’s exact test for categorical variables. Age and BMI cut-off values were determined and dichotomized using independent receiving operating curves regressing against having an RTC tear. ROC identified 57 as the age cut-off and 28 was identified as the BMI cut-off between the two groups. The “young” cohort was classified as patients <57 years and the “elderly” cohort comprised patients >57 years. The “overweight” cohort was classified as patients with BMI > 28 kg/m^2^, and the “non-overweight” cohort comprised patients with BMI < 28 kg/m^2^.

Univariate logistic regression for outcomes of RCT tear and severity of tear were conducted for predictors of interest (i.e., age and BMI). Multivariate logistic regression for outcomes of RCT and severity of tear were conducted with both age and BMI as predictors. Univariate logistic regression for outcomes of RCT tear and severity of tear with BMI as a predictor was conducted when patients were sub-analyzed in the young (<57 years) and elderly (>57 years) age cohorts. The significance level for all analyses was prespecified at 0.05. Statistical analysis was conducted in JMP Pro v.17.0.0 (Carey, NC).

## Results

Demographics

A total of 1,561 patients were included in this study. Of that, 849 (54.4%) had an RCT. The median age of patients was 58 years old (IQR 49-65). Patients with an RCT were associated with a greater median age (61, IQR 55-68) relative to those who did not have an RCT (53, p<0.0001). The median BMI for patients was 29.3 (IQR 25.8-33.7). Patients with an RCT were associated with a greater BMI (30.1, IQR 26.5-34.3) relative to those who did not have an RCT (28.3, p<0.0001). One hundred eighty-one patients (11.5%) used illicit drugs. Illicit substance use was lower in patients with an RCT (8.9% RCT vs 14.9% no RCT, p<0.0002). There was a significant association between type of work status and whether the patient had an RCT (p<0.0001). Among those who were retired, there were more with RCTs than without whereas manual vs non manual labor showed no differences. The laterality of the affected shoulder was also significantly associated with whether the patient had an RCT or not (p=0.0026); patients with RCTs had a generally higher proportion of right shoulder issues than patients without RCTs (57.8% vs. 50.7%). The RCT cohort had a significantly greater median CCI score than the no-tear group (3 vs 1, p<0.0001). Additional demographic information can be found in Table 1.

**Table 1:** Baseline demographic variable comparisons between RCT cohort and no RCT (control) cohort RCT - rotator cuff tear

Variable Name	All (n=1,561)	RCT (n=849)	No RCT (n=712)	P-value RCT vs. No RCT
Age (years), median (IQR)	58 (49-65)	61 (55-68)	53 (43-61)	<0.0001
Sex, no. (%)				
Male	688 (44.1%)	389 (45.8%)	299 (42%)	0.1296
Female	873 (55.9%)	460 (54.2%)	413 (58%)	
Race, no. (%)				
Caucasian	827 (53%)	432 (50.9%)	395 (55.5%)	0.0573
African American	357 (22.9%)	220 (25.9%)	137 (19.2%)	
Asian	48 (3.1%)	25 (2.9%)	23 (3.2%)	
Hispanic/Latino	283 (18.1%)	145 (17.1%)	138 (19.4%)	
Native American	0 (0%)	0 (0%)	0 (0%)	
Pacific Islander	8 (0.5%)	5 (0.6%)	3 (0.4%)	
Other	38 (2.4%)	22 (2.6%)	16 (2.2%)	
BMI (kg/m^2), median (IQR)	29.3 (25.8-33.7)	30.1 (26.5-34.4)	28.3 (24.9-33)	<0.0001
Tobacco Use, no. (%)				
No	781 (50%)	423 (49.8%)	358 (50.3%)	0.5957
Current	322 (20.6%)	169 (19.9%)	153 (21.5%)	
Former	458 (29.3%)	257 (30.3%)	201 (28.2%)	
Alcohol Use, no. (%)	563 (36.1%)	300 (35.4%)	263 (36.9%)	0.5226
Illicit Substance Use, no. (%)	181 (11.6%)	75 (8.9%)	106 (14.9%)	0.0002
Work Status, no. (%)				
Manual Labor	257 (16.5%)	125 (14.7%)	132 (18.5%)	<0.0001
Non-Manual Labor	373 (23.9%)	174 (20.5%)	199 (27.9%)	
Unemployed	495 (31.7%)	254 (29.9%)	241 (33.8%)	
Retired	340 (21.8%)	243 (28.6%)	97 (13.6%)	
Disabled	10 (0.6%)	5 (0.6%)	5 (0.7%)	
Unknown	86 (5.5%)	48 (57%)	38 (5.3%)	
Laterality, no. (%)				
Right	852 (54.6%)	491 (57.8%)	361 (50.7%)	0.0026
Left	693 (44.4%)	346 (40.8%)	347 (48.7%)	
Unknown	16 (1%)	12 (1.4%)	4 (0.6%)	
Severity of Tear, no. (%)				
Partial Thickness	N/A	388 (45.9%)	N/A	N/A
Full Thickness	N/A	458 (54.1%)	N/A	N/A
Number of Tendons Torn, median (IQR)	N/A	1 (1-2)	N/A	N/A
Charlson Comorbidity Index, median (IQR)	2 (1-4)	3 (1-4)	1 (1-3)	<0.0001
DVT/PE, no. (%)	21 (1.3%)	16 (1.9%)	5 (0.7%)	0.0482
MI, no. (%)	14 (0.9%)	12 (1.4%)	2 (0.3%)	0.0274
Pneumonia, no. (%)	15 (1%)	13 (1.5%)	2 (0.3%)	0.0162
30 Day Readmission, no. (%)	9 (0.6%)	7 (0.8%)	2 (0.3%)	0.1936
90 Day Readmission, no. (%)	5 (0.3%)	3 (0.4%)	2 (0.3%)	1.000

Predictors of RCT

Univariate analysis demonstrated that patients with a BMI > 28 (reference: BMI < 28, OR: 1.7, 95% CI: 1.3-2.0, p<0.0001) and age > 57 (reference: age < 57, OR: 3.7, 95% CI: 3.0-4.6, p<0.0001) are both individually associated with a greater odds of RCT. In multivariate analysis, BMI > 28 (reference: BMI < 28, aOR 1.6, 95% CI: 1.3-1.9, p<0.0001) and age > 57 years old (reference: age<57, aOR 3.7, 95% CI: 3.0-4.5, p<0.0001) are significant predictors of RCT (Table 2).

**Table 2:** Unadjusted (univariate) and adjusted (multivariate) odds ratios predicting outcome of RCT and severity of tear RCT - rotator cuff tear

Variable	Odds ratio (95% CI)	P-value	Adjusted odds ratio (95% CI)	P-value
For Outcome RCT vs. No Tear				
BMI (kg/m^2^)				
<28	Reference		Reference	
>28	1.6512 (1.3480-2.0227)	<0.0001	1.5547 (1.2559-1.9247)	<0.0001
Age (years)				
<57	Reference		Reference	
>57	3.7414 (3.0304-4.6193)	<0.0001	3.6645 (2.9646-4.5295)	<0.0001
For Outcome Full Thickness vs. Partial Thickness Tear Severity				
BMI (kg/m^2^)				
<28	Reference		Reference	
>28	1.3945 (1.0520-1.8483)	0.0207	1.3943 (1.0503-1.8511)	0.0215
Age (years)				
<57	Reference		Reference	
>57	1.5919 (1.1820-2.1441)	0.0022	1.5918 (1.1808-2.1459)	0.0023

When stratifying patients by age (with the cut-off of 57 years), univariate analyses demonstrated that BMI > 28 was significantly associated with an increased odd of RCT (Table 3). The odds ratio of higher BMI (BMI > 28 vs. BMI < 28) for the outcome of RCT tear was greater in the younger age group (adjusted odds ratio (aOR) 1.8, 95% CI: 1.3-2.4, p<0.0004) in comparison to the older age group (aOR 1.4, 95% CI: 1.1-1.9, p=0.03, Table 3).

**Table 3:** Odds ratios of BMI predictor for outcome RCT and severity by age sub-cohorts RCT - rotator cuff tear

Variable	Odds ratio (95% CI) for RCT tear vs. no tear	P-value	Odds ratio (95% CI) for full thickness vs. partial thickness tear	P-value
Age < 57 years				
BMI (kg/m^2^)				
<28	Reference		Reference	
>28	1.7731 (1.2910-2.4353)	0.0004	1.7418 (1.0278-2.9519)	0.0392
Sex				
Male	Reference		Reference	
Female	0.7314 (0.5355-0.9990)	0.0493	0.8433 (0.5114-1.3906)	0.5042
Race				
Caucasian	Reference		Reference	
African American/Black	2.2032 (1.4978-3.2409)	<0.0001	1.3858 (0.7697-2.4949)	0.2768
Asian	1.7160 (0.7289-4.0399)	0.2164	2.6374 (0.6245-11.1299)	0.1871
Hispanic/Latino	1.1271 (0.7427-1.7105)	0.5739	0.8636 (0.4292-1.7369)	0.6809
Tobacco Use				
No	Reference		Reference	
Current	1.0672 (0.7303-1.5596)	0.7368	0.8256 (0.4475-1.5228)	0.5394
Former	1.0706 (0.7352-1.5592)	0.7220	0.9140 (0.5009-1.6677)	0.7695
Alcohol Use				
No	Reference		Reference	
Yes	1.0037 (0.7290-1.3818)	0.9822	0.9912 (0.5936-1.6552)	0.9731
Illicit Drug Use				
No	Reference		Reference	
Yes	0.6729 (0.4337-1.0441)	0.0772	0.6393 (0.2995-1.3658)	0.2476
Work Status				
Manual Labor	Reference		Reference	
Non-Manual Labor	0.5930 (0.3836-0.9166)	0.0187	0.8992 (0.4381-1.8456)	0.7721
Unemployed	0.8467 (0.5686-1.2610)	0.4128	1.4649 (0.7801-2.7508)	0.2351
Retired	2.0956 (0.3409-12.8831)	0.4246	0.7027 (0.0609-8.1598)	0.7787
Disabled	0.3493 (0.4883-1.7766)	0.3516	N/A	N/A
Laterality				
Right	Reference		Reference	
Left	0.6624 (0.4826-0.9093)	0.0108	1.2111 (0.7253-2.0221)	0.4641
CCI	1.0562 (0.9371-1.1905)	0.3705	1.0085 (0.8058-1.2622)	0.9412
Age > 57 years				
BMI (kg/m^2^)				
<28	Reference		Reference	
>28	1.3918 (1.0410-1.8608)	0.0257	1.2719 (0.9077-1.7821)	0.1623
Sex				
Male	Reference		Reference	
Female	0.8634 (0.6463-1.1534)	0.3201	0.9495 (0.6846-1.3170)	0.7564
Race				
Caucasian	Reference		Reference	
African American/Black	1.2424 (0.8647-1.7850)	0.2405	1.9547 (1.2944-2.9512)	0.0014
Asian	0.7454 (0.3277-1.6957)	0.4835	2.7167 (0.8477-8.7065)	0.0926
Hispanic/Latino	1.1210 (0.7475-1.6812)	0.5806	1.7500 (1.0965-2.7930)	0.0190
Tobacco Use				
No	Reference		Reference	
Current	1.1861 (0.7880-1.7855)	0.4134	0.8663 (0.5516-1.3606)	0.5332
Former	1.0507 (0.7623-1.4483)	0.7626	0.8050 (0.5581-1.1611)	0.2458
Alcohol Use				
No	Reference		Reference	
Yes	0.9632 (0.7141-1.2993)	0.8063	0.8800 (0.6256-1.2380)	0.4630
Illicit Drug Use				
No	Reference		Reference	
Yes	0.7306 (0.4381-1.2182)	0.2288	1.0960 (0.5787-2.076)	0.7785
Work Status				
Manual Labor	Reference		Reference	
Non-Manual Labor	1.2552 (0.7409-2.0892)	0.4088	0.7969 (0.4219-1.5054)	0.4843
Unemployed	1.3153 (0.8004-2.1615)	0.2795	0.9655 (0.5258-1.7731)	0.9099
Retired	1.6399 (1.0176-2.6426)	0.0422	2.0489 (1.1378-3.6716)	0.0166
Disabled	2.5965 (0.2792-24.1478)	0.4017	2.8965 (0.2841-29.5328)	0.3693
Laterality				
Right	Reference		Reference	
Left	0.7879 (0.5910-1.0504)	0.1042	1.0075 (0.7236-1.4028)	0.9646
CCI	1.0858 (1.0049-1.1733)	0.0374	1.0501 (0.9650-1.1426)	0.2572

Predictors of RCT tear severity

Univariate analysis demonstrated that patients with a BMI > 28 (reference: BMI < 28, OR: 1.4, 95% CI: 1.1-1.8, p=0.0207) and age > 57 (reference: age < 57, OR: 1.6, 95% CI: 1.2-2.1, p=0.0022) are both individually associated with a greater odd of increased severity of RCT, defined by a comparison of partial thickness versus full thickness RCT within patients. In multivariate analysis, BMI > 28 (reference: BMI < 28, aOR 1.4, 95% CI: 1.1-1.9, p=0.0215) and age > 57 years old (reference: age<57, aOR 1.6, 95% CI: 1.2-2.1, p=0.0023) are significant predictors of a full thickness tear.

Differences in demographics in patients less than 57 years with and without RCT

Univariate analyses demonstrated that BMI > 28 is significantly associated with increased odds of a full thickness tear in our younger patients (reference: BMI < 28, aOR: 1.7, 95% CI: 1.3-2.4, p=0.0392) when compared to no RCT or partial thickness tears, respectively. Female patients are associated with decreased odds of RCT within this cohort (aOR: 0.7, 95% CI: 0.5-1, p=0.0493). African American patients have increased odds of an RCT relative to White patients within this cohort (aOR: 2.2, 95% CI: 1.5-3.2, p<0.0001). Patients who participated in non-manual labor have decreased odds of RCT (reference: manual labor, aOR: 0.6, 95% CI: 0.4-0.9, p=0.0187).

Differences in demographics in patients greater than 57 years with and without RCT

Univariate analyses in our older patient cohort demonstrated that patients with BMI > 28 have increased odds of RCT relative to patients with BMI < 28 within this cohort (aOR: 1.4, 95% CI: 1.0-1.9, p=0.0392). African American (aOR: 2.0, 95% CI: 1.3-3.0, p=0.0014) and Hispanic patients (aOR: 1.8, 95% CI: 1.1-2.8, p=0.019) are at increased odds of a full-thickness tear compared to White patients. Retired patients have increased odds of RCT (aOR: 1.6, 95% CI: 1.3-3.0, p=0.0422) and full thickness tears (aOR: 2.0, 95% CI: 1.1-3.7, p=0.0166) relative to patients who work manual labor. Patients with greater CCI are associated with increased odds of RCT (aOR: 1.1, 95% CI: 1.0-1.2, p=0.0374, Table 3).

## Discussion

RCTs are a highly prevalent condition among patients seeking care from orthopedic surgeons for shoulder complaints [2]. They are thought to have a multifaceted etiology, and thus, current studies aim to delineate potential risk factors associated with developing a tear. Additionally, it has been theorized that there may be variation among the already identified factors between younger and older patients who present with an RCT [10]. As a result, this study aims to identify factors associated with an increased risk for RCT and determine whether there were any notable differences between younger and older patients treated at our institution. This study hypothesized that patients with an RCT would demonstrate differences in their demographic characteristics compared to those without RCT. Initial independent ROC curves set our cut-off age and BMI values at 57 years and BMI at 28. From there, we determined our groups, and it was observed that the CCI score, BMI, sex, race, and work status were all factors that variably affected a patient's risk of experiencing an RCT, with different factors carrying more influence on outcomes within the groups.

Our baseline demographic analysis found that patients with an RCT were associated with a higher CCI score. This sensitive assessment tool, which takes into account medical comorbidities, is designed to predict long-term risk of mortality [11] and the difference observed may suggest an interplay between RCT and chronic medical conditions. Among all age groups, there were similar relationships observed in relation to greater CCI scores in our RCT cohort. Further analysis into individual variables showed that increased age and BMI were associated with patients who had RCTs compared to those who did not which aligns with current literature. A review from Tashjian et al. reported that more than half of individuals in their 80s have RCTs with increasing age, further correlating with increased tear size and negatively affecting postoperative healing [4,12]. Contrary to current literature that illustrates smoking history as a predisposing factor, our retrospective analysis found that smoking was not significantly different between the RCT and no RCT cohorts [4]. Similarly, a meta-analysis from Zhao et al. reported that from an analysis of five studies, smoking did not increase the risk of full-thickness RCTs [1].

Univariate analysis demonstrated that patients with a BMI greater than 28 had greater odds of experiencing an RCT. Multivariate analysis supported these results as BMI remained a significant predictor for RCTs. We also found that younger RCT patients had higher odds of having a higher BMI than the older cohort. Similarly, Gumina et al. reported that over 60% of their patient cohort, all younger than 50 years of age, had a BMI greater than 25, and 25% had a BMI classified as obese [13]. Generally, increased BMI is a well-known comorbidity that contributes to poorer patient outcomes. While the biology of increased body fat percentage in RCTs is still being explored, there is literature that demonstrates how the accumulation of fatty tissue decreases elasticity within the shoulder and can ultimately place the rotator cuff at a greater risk of tearing while also hindering the ability to heal after repair [14]. Furthermore, within those patients younger than 57 years of age, BMI was a significant predictor for the presence of RCT and increased severity of RCTs. It was observed that younger patients with a BMI greater than 28 were at a higher risk of experiencing a full-thickness RCT than a partial-thickness RCT. Our results align with others reported by Weng et al. [9] and Macchi et al. [8]. Fatty infiltration, anteroposterior tear size, and retraction size of RCTs were all greater in patients who were overweight or obese compared to patients with a normal BMI [9].

Similar to BMI, univariate and multivariate analysis of age demonstrated that patients older than 57 were associated with greater odds of experiencing RCTs. Age-related degenerative changes of the rotator cuff are a known causative agent of RCTs. While the exact prevalence of tears can differ depending on the primary form of occupation and demographic of a community, a general increasing trend of prevalence in RCTs with age is commonly observed [2].

As mentioned previously, there is a paucity of literature available exploring whether there are differences between age cohorts in suspected RCT risk factors. In our analyses, we found differences in sex, race, and work status when considering their effect on RCTs in our old vs young age cohorts. Female sex was only found to decrease the odds of an RCT in our younger cohort. With regard to race, our age cohorts differed in the effect of those identifying as Hispanic on the likelihood of RCT. Both cohorts had a higher rate of tears in African Americans, but the older population also had a higher incidence of tears in our Hispanic patients while the younger cohort did not. According to a report published by the United States Bureau of Labor Statistics, employed African American and Hispanic men were more likely when compared to White and Asian men to work within production, transportation, and material moving occupations [15]. These physically intensive careers are widely documented to increase one's risk of experiencing an RCT, especially if repeatedly engaging in overhead motions and heavy lifting [16]. A retrospective review of patients conducted by Ko et al. confirmed that high physical work level and atrophy of rotator cuff muscles were both independent risk factors that increased the risk of one experiencing an RCT [17]. Work status was another variable that differed between our age cohorts. Our younger cohort had increased odds of RCT if they engaged in manual labor whereas our older cohort had an increased risk of RCT if they were retired. This is likely a result of a higher risk of RCT with increasing age which also corresponds to a higher likelihood of being retired.

The present study had several limitations. First, it was a retrospective review and thus was subject to common biases of retrospective studies. Causality cannot be inferred from these results. Second, within our study, we utilized the traditional measure of BMI to classify individuals as overweight or not. However, BMI has several deficiencies as a measure of fat as it is an indirect measure of body fat compared to other methods, such as bioelectrical impedance or skinfold calipers to measure body fat percentage [18]. While BMI will sufficiently measure most of the patient population, some individuals will be inaccurately classified due to atypical weight-to-height ratio from greater muscle mass. When replicating this study, body fat percentage would be a more accurate measure of fatty tissue accumulation and allow for more significant conclusions to be made about the relationship between excess weight and RCTs. Additionally, due to the retrospective nature of this study, we could not accurately account for known previous shoulder conditions or injuries. Further studies are needed to explore the impact of potential risk factors for RCTs in the younger cohort, as RCTs occur less frequently in this cohort.

## Conclusions

In conclusion, our analyses identified age and BMI cut-off values where the risk of sustaining an RCT seemed to increase. From there, we observed that BMI, sex, race, and occupation type (manual labor) were statistically significant risk factors for RCTs in younger patients. Within the older cohort, we similarly observed that BMI, race, and occupation type (being retired) were statistically significant indicators of increasing the odds of experiencing an RCT. Our only differences between the two groups were that younger patients tended to be male, manual laborers, and African American and had higher odds of having a high BMI, while older patients showed no difference in sex, were more likely to be retired, and had a higher rate of Hispanic patients. We feel that our findings contribute to the growing literature on specific rotator cuff tear risk factors overall as well as stratified by age cohorts. By recognizing these age-specific factors, physicians may be able to diagnose and treat patients who are identified as being at a higher risk of experiencing RCTs more accurately.
